# 0895. Identification and validation of a mirna as a diagnostic biomarker of diffuse alveolar damage in an animal model of acute lung injury and adult respiratory distress syndrome in mechanically ventilated patients

**DOI:** 10.1186/2197-425X-2-S1-O21

**Published:** 2014-09-26

**Authors:** P Cardinal-Fernández, A Ferruelo, N Rego, Y Rojas, A Ballén-Barragán, R Granados, C Jaramillo, E Lopez-Hernández, L Martínez-Caro, N Nin, R Herrero, MA de la Cal, A Esteban, JA Lorente

**Affiliations:** Centro de Investigación Biomédica En Red de Enfermedades RespiratoriaS (CIBERES), Getafe, Spain; Hospital Universitario de Getafe, Intensive Care Service and Burn Unit, Getafe, Spain; Institut Pasteur de Montevideo, Unidad de Bioinformatica, Montevideo, Uruguay; Hospital Universitario de Getafe, Pathology Department, Getafe, Spain; Hospital Universitario de Getafe, Getafe, Spain; Hospital de Torrejon, Intensive Care Service, Madrid, Spain; Hospital Español, Intensive Care Service, Montevideo, Uruguay; Universidad Europea de Madrid, Madrid, Spain

## Objective

To discover a miRNA with diagnostic characteristics for diffuse alveolar damage (DAD) in an animal of acute lung injury (ALI) and in patients with the ARDS.

## Methods

Male rats (325-372 gr) underwent mechanical ventilation for 2.5 h with V_T_=9 ml/kg + PEEP=5 cm H_2_O (low V_T_, LV, n=10); or V_T_=25 ml/kg + PEEP=0 cm H_2_O (high V_T_, HV, n=19) (11 of 19 developed DAD at histological examination). Whole miRNA expression (RNA-seq-Single Read, 72 cycles, Illumina GaIIx) was analyzed in lung parenchyma. miRNA expression in LV vs. HV and in HV-DAD vs. HV-no-DAD was compared. We used a data mining strategy to prioritize the most relevant miRNA within the miRNAs differentially expressed. Prioritized miRNAs were validated in (1) serum from the same group of rats (RT-PCR); (2) human lung tissue (preserved at -80º after sampling) from autopsies of patients with ARDS (RT-PCR and *in situ* hybridization) (n=20); (3) human serum from mechanically ventilated patients obtained during the first 24 hours of ICU admission (n=66, 14 nonsurvivors). A p value < 0.05 was considered statistically significant. Results are median (IQR), and odds ratio (OR [95% confidence interval]). RT-PCR results were expressed x10^-4^. Categorical and continuous variables were compared with χ2 and Mann-Whitney, respectively. Predictive multivariate logistic analysis was used to identify independent risk factors. The area under ROC curve (AUROC, mean±SEM) was used to assess the discriminatory capacity. Multiple-comparison was adjusted by FDR. The local Ethics Committee approved this study.

## Results

19 miRNAs were differentially expressed **in rat lungs** between the 3 groups, one of them (herein named miRNA A) containing the information to classify animals in the 3 groups (97% correct classification in the original dataset, and 86% in 5 fold cross-validation analysis). **In rat serum**, miRNA A expression differed in the 3 groups (p=0.045) (AUROC for the presence of DAD 0.76 [0.521-1.000], p=0.036 for the difference with the line of identity). miRNA expression **in human serum** was associated with mortality. Variables in multivariate analyses for the preediction of mortality were: SAPS II score (OR 1.05 [1.01-1.10], p=0.02), and miRNA A expression (OR 1.06 [1.01-1.12; p=0.04) (AUROC for the logistic model 0.84 [0.73-0.96]; for SAPSII 0.76 [0.60-0.92] and for miRNA A 0.76 [0.63-0.89]). miRNA A was more expressed **in lung tissue** from 39 patients with the diagnosis of ARDS than in patients without ARDS (p< 0.01). In **human lung parenchyma** miRNA A was detected (RT-PCR), localized in alveolar type II cells, macrophages and interstitium (*in situ* hybridization).

## Conclusions

We have identified one miRNA associated with DAD in an animal model of ALI, that is expressed in human lung tissue, and whose expression in human serum correlates with mortality and with the diagnosis of ARDS.

